# Risk Factors and Predictors of Mechanical Ventilation in Neonates With Meconium Aspiration Syndrome: A Retrospective Study at King Salman Armed Forces Hospital, Tabuk, Saudi Arabia

**DOI:** 10.7759/cureus.88486

**Published:** 2025-07-22

**Authors:** Abdulaziz A Bedaiwi, Muhanned Amawi, Wegdan Mawlana

**Affiliations:** 1 Pediatrics, King Salman Armed Forces Hospital, Tabuk, SAU; 2 Pediatrics and Neonatology, King Salman Specialized Hospital, Taif, SAU

**Keywords:** cesarean section, mechanical ventilation, meconium aspiration syndrome, meconium-stained amniotic fluid, neonatal respiratory distress, persistent pulmonary hypertension of the newborn, surfactant therapy

## Abstract

Background

Meconium aspiration syndrome (MAS) is a critical neonatal condition predominantly associated with term and post-term pregnancies, characterized by the aspiration of meconium-stained amniotic fluid (MSAF) leading to severe respiratory distress. Despite advances in obstetric and neonatal care, MAS remains a significant cause of neonatal morbidity, particularly in settings with limited access to advanced neonatal care.

Objectives

This study aimed to evaluate the prevalence of mechanical ventilation and explore potential predictors for respiratory support in neonates diagnosed with MAS at King Salman Armed Forces Hospital (KSAFH) in Tabuk, Saudi Arabia, from 2020 to 2024.

Methods

A retrospective cross-sectional analysis was conducted on 329 full-term and post-term neonates diagnosed with MAS. Data were extracted from medical records, including demographic details, clinical characteristics, treatment modalities, and outcomes. Statistical analyses were performed to identify correlations between these variables and the need for mechanical ventilation.

Results

The study revealed a slight male predominance (193, 58.7%) among neonates with MAS, with a mean gestational age of 39 weeks and a mean birth weight of 3.19 kg (SD = 1.67). A significant association was observed between cesarean section (CS) deliveries and the administration of surfactant therapy (p = 0.019). Mechanical ventilation was required in 30 (9.1%) of the neonates, with seven (2.1%) requiring high-frequency oscillatory ventilation (HFOV). The most common pregnancy complication associated with MAS was a non-reassuring fetal heart rate, affecting 162 (49.2%) of cases. However, no significant associations were found between maternal medical history, pregnancy complications, and the need for mechanical ventilation. The average neonatal intensive care unit (NICU) stay was 5.33 days (SD = 4.37), with persistent pulmonary hypertension of the newborn (PPHN) observed in 14 (4.3%) of cases.

Conclusions

MAS remains a challenging condition in neonatal care, with significant respiratory morbidity necessitating advanced respiratory support. The study underscores the importance of tailored management strategies, particularly in neonates delivered via CS. Further research is needed to refine treatment protocols and improve long-term outcomes for neonates with MAS.

## Introduction

Meconium aspiration syndrome (MAS) remains a significant neonatal condition, particularly associated with term and post-term pregnancies, where the presence of meconium-stained amniotic fluid (MSAF) can lead to severe respiratory distress. This syndrome is characterized by the aspiration of meconium into the infant's lungs either during delivery or, less commonly, in utero. The pathophysiology involves airway obstruction, chemical pneumonitis, and inactivation of pulmonary surfactant, which together contribute to varying degrees of hypoxia and respiratory failure [1].

The incidence of MAS has shown a notable decline in recent years, primarily due to improved obstetric practices such as the prevention of post-term pregnancies and enhanced fetal monitoring during labour. Despite this, MAS continues to present clinical challenges, particularly in resource-limited settings where access to advanced neonatal care may be constrained [2]. Risk factors for MAS include thick meconium, a pathological cardiotocographic trace, fetal acidosis, caesarean section, in addition to maternal conditions such as hypertension and diabetes, as well as intrauterine growth restriction and prolonged labour, which contribute to fetal distress and the subsequent passage of meconium [3,4].

In clinical practice, the management of MAS has evolved significantly. The historical approach of routine endotracheal suction for non-vigorous neonates has been largely abandoned in favor of more evidence-based practices. Current guidelines emphasize supportive care, including oxygen supplementation, surfactant therapy, and in severe cases, the use of extracorporeal membrane oxygenation (ECMO) to manage refractory respiratory failure [5]. However, the optimal management of MAS continues to be debated, with ongoing research aimed at refining treatment protocols and improving long-term outcomes for affected neonates.

Despite advancements in treatment, MAS remains a significant contributor to neonatal morbidity, particularly due to its association with persistent pulmonary hypertension of the newborn (PPHN) [6]. This underscores the need for continued vigilance and research into preventive and therapeutic strategies to mitigate the impact of MAS on neonatal health.

There is currently a lack of data regarding the rate of mechanical ventilation required for neonates diagnosed with MAS admitted to the neonatal intensive care unit (NICU) at King Salman Armed Forces Hospital (KSAFH) in Tabuk. Consequently, this study aims to identify the prevalence of mechanical ventilation among these neonates and to detect potential predictors or risk factors that may influence the selection of treatment options for MAS.

## Materials and methods

This study was conducted as a retrospective cross-sectional analysis in the NICU at KSAFH in Tabuk. The research focused on neonates diagnosed with MAS who were admitted to the NICU from January 2020 to January 2024. The study aimed to identify correlations between various demographic and clinical characteristics and the selection of the treatment provided.

Study design and population

A total of 329 full-term and post-term neonates diagnosed with MAS were included in this study. The inclusion criterion was the diagnosis of MAS during the study period. Preterm neonates, in addition to term and post-term babies who were discharged home within 24 hours or not admitted to NICU, and any baby with a major congenital anomaly were excluded from this study. The exclusion of neonates discharged within 24 hours or not admitted to the NICU was based on the need to focus on cases of MAS that required significant medical intervention and monitoring. This approach ensures that the study results are applicable to the population most at risk for severe MAS outcomes, though it may limit the generalizability of the findings to all neonates with MAS. A purposive sampling technique was employed to select the study population. Data for this study were extracted from the patients' medical records, ensuring a comprehensive collection of demographic details, treatment modalities, and clinical outcomes.

Data collection and variables

Demographic and treatment-related data were meticulously gathered from the medical records. Variables collected included birth weight, gestational age, APGAR scores at various time intervals, time of intubation, highest mean airway pressure (MAP), extubation time, and length of stay in the NICU. Additionally, categorical variables such as gender, mode of delivery, presence of MSAF, and respiratory support types were recorded.

Statistical analysis

Data were entered into IBM SPSS Statistics for Windows, Version 25 (Released 2017; IBM Corp., Armonk, New York, United States) for statistical analysis. Descriptive statistics were used to summarize demographic and treatment-related characteristics, which were presented in both tabular and graphical formats. To explore potential associations between demographic/clinical characteristics and treatment options, Chi-square tests and Fisher's exact tests were conducted for categorical variables. For continuous variables, independent sample t-tests and analysis of variance (ANOVA) were applied to determine differences in means between groups. A p-value of ≤0.05 was considered statistically significant.

## Results

The study cohort comprised 329 neonates diagnosed with MAS, with a slight male predominance, as 58.7% (193 neonates) were male. The mean birth weight was 3.19 kg (SD = 1.67), and the mean gestational age was 39 weeks (SD = 1.30). APGAR scores were recorded at 1, 5, and 10 minutes, with mean scores of 6, 8, and 9, respectively. The mean time of intubation was 2.14 hours (SD = 4.09), and average extubation time was 3.66 days (SD = 4.85). The length of stay in the NICU ranged from 2 to 44 days, with an average stay of 5.33 days (SD = 4.37) (Table 1).

**Table 1:** Neonatal Characteristics and Outcomes Descriptive statistics for birth weight, gestational age, APGAR scores (1 and 5 minutes), intubation time, mean airway pressure (MAP), extubation time, and neonatal intensive care unit (NICU) length of stay.

Item		N	Minimum	Maximum	Mean	Std. Deviation
Birth weight (kg)		329	1.71	5.6	3.10	0.54
Gestational age (week)		329	35	42	39.38	1.30
APGAR score	At 1 min	329	1	9	6.39	1.93
	At 5 mins	329	2	9	7.91	1.22
	At 10 mins	34	4	9	7.18	1.14
	At 20 mins	4	4	8	6.25	1.71
Time of intubation (hours)		37	1	24	2.14	4.09
Highest MAP (mean)		36	5	25	12.87	5.93
Extubating time (day)		32	1	21	3.66	4.85
Length of stay		329	2	44	5.33	4.37

Regarding the mode of delivery, most neonates (166, 50.5%) were delivered via cesarean section (CS), followed by 128 (38.9%) through spontaneous vaginal delivery (SVD), and a smaller proportion (35, 10.6%) were born through assisted delivery methods, such as forceps or vacuum extraction (Table 2).

**Table 2:** Clinical Characteristics and Interventions in Neonates With Meconium Aspiration Syndrome (MAS) Summary of demographic data, mode of delivery, clinical interventions (e.g., mechanical ventilation, surfactant therapy), and complications observed in neonates diagnosed with MAS. CS: cesarean section; SVD: spontaneous vaginal delivery; CPAP: continuous positive airway pressure; PPHN: persistent pulmonary hypertension of the newborn

Variables	Categories	Number	Percentage (%)
Gender	Male	193	58.7
	Female	136	41.3
Mode of delivery	Assisted delivery	35	10.6
	CS	166	50.5
	SVD	128	38.9
Recusation	Positive pressure ventilation	90	27.4
	Intubation and mechanical ventilation	32	9.7
Type of respiratory support on admission	CPAP	171	52
	Non-invasive ventilation	103	31.3
	Mechanical ventilation	30	9.1
	High frequency oscillatory ventilation	7	2.1
	Nasal canula	18	5.5
Mode of ventilation	Conventional	30	81.1
	High‐frequency oscillation	7	18.9
Servanta given	Given	25	7.6
Inhaled nitric oxide	Given	10	3
Inhaled tropes	Given	12	3.6
Cooling thereby	Given	6	1.8
Complications	PPHN	14	4.3
	Infection	11	3.3

**Figure 1:**
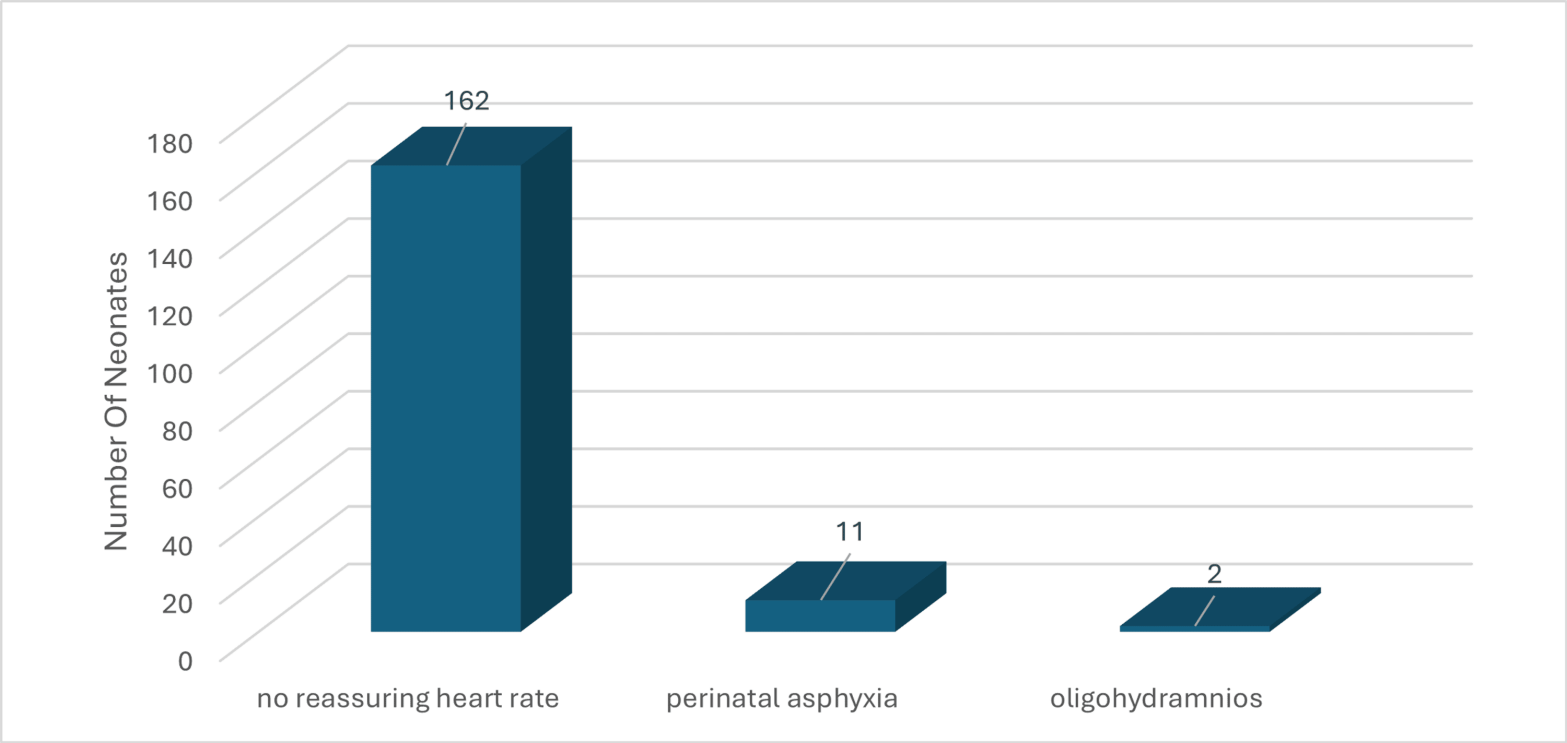
Pregnancy Complications in Neonates With Meconium Aspiration Syndrome (MAS) Bar chart displaying the frequency of specific pregnancy complications in mothers of neonates diagnosed with MAS, including non-reassuring heart rate, perinatal asphyxia, and oligohydramnios.

No significant association was found between gender and treatment selection (p > 0.05). However, a statistically significant association was identified between the mode of delivery and the administration of Servanta (p = 0.019), with neonates delivered via CS being more likely to receive Servanta. No significant associations were found between the presence of MSAF, respiratory distress, and treatment selection. Additionally, the study found no significant differences in outcomes based on the type of resuscitation or respiratory support provided at admission. Despite various maternal histories and pregnancy complications, including maternal diabetes mellitus (DM) and hypothyroidism, no significant associations with clinical outcomes were identified (p > 0.05) (Figure 1 and Table 3).

**Table 3:** Independent Sample t-Test Analysis of Clinical Outcomes Based on Maternal History and Pregnancy Complications This table presents the results of independent sample t-tests comparing various clinical outcomes (time of intubation, highest FiO_2_ requirement, highest MAP, extubation time, and length of stay) in relation to the presence or absence of maternal history and pregnancy complications in neonates with MAS. The table includes mean values, standard deviations (SD), t-statistics, and p-values for each comparison, indicating whether differences between groups are statistically significant. MAP: mean airway pressure; MAS: meconium aspiration syndrome

Item	Maternal History	Mean	SD	T Statistics	P-value	Complications During Pregnancy	Mean	SD	T Statistics	P-value
Time of intubation (hours)	Yes	3.88	8.13	1.376	0.178	Yes	1.45	1.92	-1.235	0.225
	No	1.66	1.97			No	3.13	5.98		
Highest FiO_2_ requirement	Yes	41.88	25.49	0.12	0.905	Yes	40.23	21.41	-0.256	0.799
	No	40.80	21.83			No	42.13	24.10		
Highest MAP (mean)	Yes	10.71	5.22	1.171	0.250	Yes	15.79	16.64	0.435	0.666
	No	13.48	6.07			No	13.82	6.23		
Extubating time (day)	Yes	6.57	7.85	1.87	0.071	Yes	4.33	5.70	1.095	0.282
	No	2.84	3.44			No	2.36	2.25		
Length of stay	Yes	5.86	4.93	1.068	0.286	Yes	5.53	4.41	0.845	0.399
	No	5.20	4.23			No	5.12	4.34		

**Figure 2:**
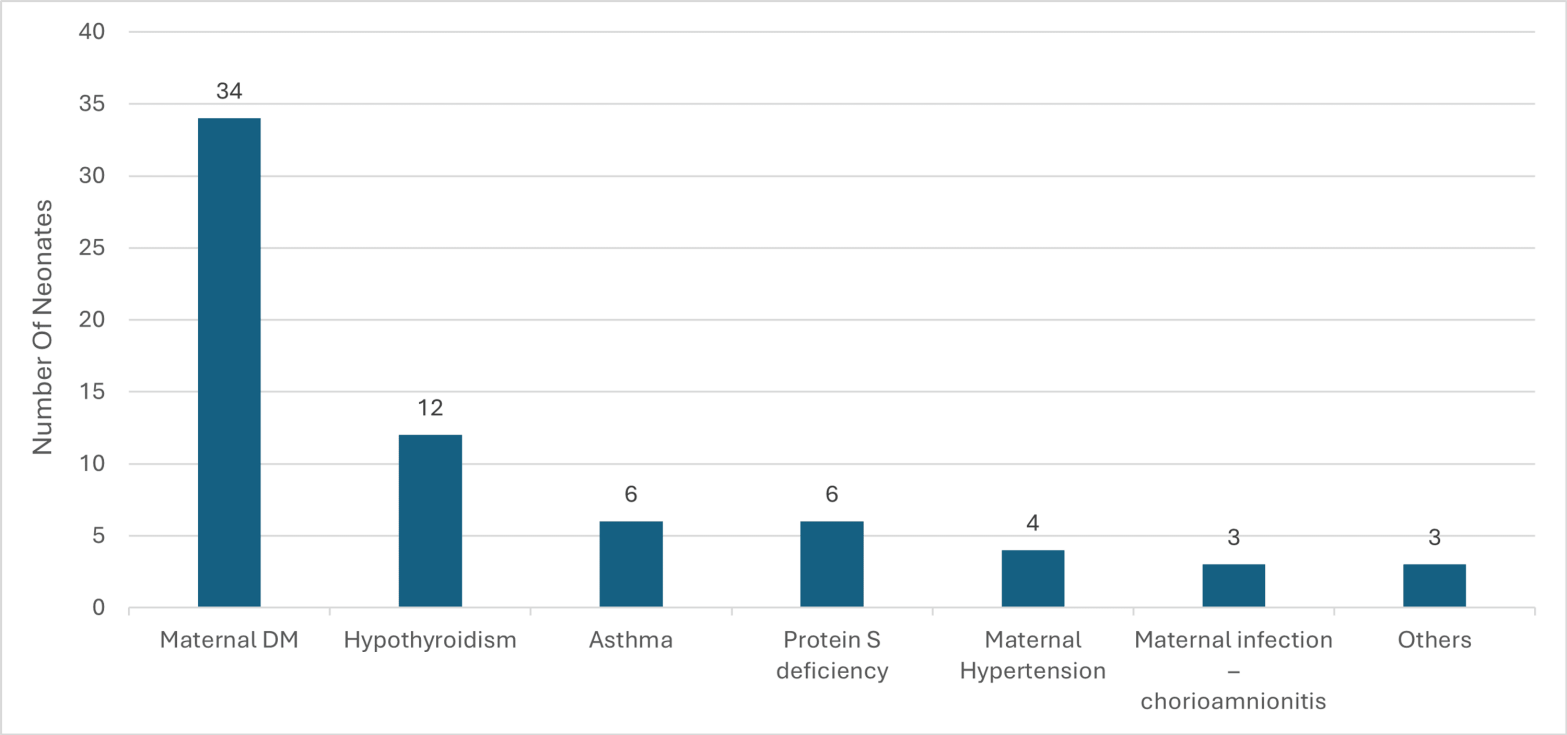
Distribution of Maternal History in Neonates With Meconium Aspiration Syndrome (MAS) Bar chart illustrating the frequency of maternal medical conditions in neonates diagnosed with MAS. Conditions include maternal diabetes mellitus (DM), hypothyroidism, asthma, protein S deficiency, maternal hypertension, and maternal infection (chorioamnionitis).

Independent sample t-tests and ANOVA revealed no statistically significant differences in birth weight, gestational age, time of intubation, extubation time, highest FiO_2_, highest MAP, and length of stay among different demographic or clinical variables, such as mode of delivery and maternal history (p > 0.05) (Figure 2). Chi-square analysis confirmed no significant associations between the demographic or medical characteristics of the patients and the outcomes of MAS, except for the significant relationship between the mode of delivery and Servanta administration. This finding suggests that while most demographic and clinical factors did not significantly affect outcomes, the mode of delivery influenced the treatment strategy (Tables 4-5).

**Table 4:** ANOVA Analysis of Clinical Outcomes Based on Mode of Delivery This table shows the results of an analysis of variance (ANOVA) comparing various clinical outcomes, such as time of intubation, highest FiO_2_ requirement, highest MAP, extubation time, and length of stay, across different modes of delivery (assisted delivery, cesarean section (CS), and spontaneous vaginal delivery (SVD)) in neonates with MAS. The table includes mean values, standard deviations (SD), F statistics, and p-values to determine if there are statistically significant differences between the groups. MAS: meconium aspiration syndrome; MAP: mean airway pressure

Item	Mode of Delivery	Mean	SD	F Statistics	P-value
Time of intubation (hours)	Assisted delivery	5.5	6.4	0.726	0.491
	CS	2.0	4.7		
	SVD	1.7	1.8		
Highest FiO_2_ requirement	Assisted delivery	32.5	10.6	0.149	0.862
	CS	41.3	22.4		
	SVD	41.8	24.4		
Highest MAP (mean)	Assisted delivery	13.5	9.2	0.270	0.765
	CS	12.3	5.6		
	SVD	13.9	6.5		
Extubating time (day)	Assisted delivery	1.5	0.7	0.2	0.82
	CS	3.8	5.2		
	SVD	3.8	4.8		
Length of stay	Assisted delivery	4.4	1.7	1.267	0.283
	CS	5.6	4.4		
	SVD	5.2	4.8		

**Table 5:** ANOVA Analysis of Birth Weight and Gestational Age by Resuscitation and Respiratory Support This table presents the results of an analysis of variance (ANOVA) comparing the mean birth weight and gestational age of neonates across different resuscitation methods and types of respiratory support on admission in cases of MAS. The table includes mean values, standard deviations (SD), F statistics, and p-values to evaluate if there are statistically significant differences between the groups. CPAP: continuous positive airway pressure; MAS: meconium aspiration syndrome

Item		Birth weight (kg)				Gestational age (week)			
		Mean	SD	F Statistics	P-value	Mean	SD	F Statistics	P-value
Type of respiratory support on admission	CPAP	3.09	0.48	0.676	0.609	39.41	1.19	1.006	0.405
	Noninvasive ventilation	3.40	2.87			39.44	1.40		
	Mechanical ventilation	2.98	0.75			39.07	1.53		
	High frequency oscillatory ventilation	3.26	0.56			38.71	1.38		
	Nasal canula	3.18	0.63			39.5	1.30		

## Discussion

MAS is a significant cause of respiratory distress in term and post-term neonates. It is characterized by respiratory failure in neonates born through MSAF, with symptoms that are not attributable to other causes and are accompanied by distinctive radiological findings [7,8]. The diagnosis of MAS is generally determined by several key criteria: respiratory distress in a newborn delivered through MSAF, the necessity for oxygen to keep transcutaneous saturation above 92%, the initiation of oxygen therapy within the first two hours after birth, continued for a minimum of 12 hours, and the exclusion of any congenital malformations affecting the airways, lungs, or heart [9]. The present retrospective cross-sectional study, conducted at the NICU of KSAFH in Tabuk from 2020 to 2024, focused on neonates diagnosed with MAS to evaluate demographic and clinical characteristics, treatment options, and the prevalence of mechanical ventilation among this population.

The study included 329 full-term and post-term neonates diagnosed with MAS, revealing critical insights into the clinical management of this condition and its associated outcomes. This discussion synthesizes the findings from the current study with existing literature, providing a comprehensive overview of MAS in this hospital setting.

The cohort exhibited a slight male predominance, with 58.7% of the neonates being male, which aligns with some studies [10] but contrasts with the findings of Espinheira et al., who reported a 55.6% female predominance in their study [11]. The mean gestational age was 39.38 weeks, and the mean birth weight was 3.19 kg, consistent with the known association of MAS with term and post-term neonates, where the likelihood of MSAF increases due to fetal stress in late pregnancy stages. The high prevalence of MSAF in this cohort (96.7%) underscores its role as a critical indicator for MAS [12].

Respiratory distress was a prevalent complication, observed in all cases; however, 1.2% was mild and resolved within hours, highlighting the severe impact of MAS on neonatal respiratory function and the critical need for immediate and effective respiratory support post-delivery [5]. The mode of delivery also played a significant role in the clinical outcomes of MAS. CS deliveries were more common (50.5%) compared to SVD (38.9%) and assisted deliveries (10.6%). The significant association between the mode of delivery and the administration of surfactant therapy (Servanta) (p = 0.019) may suggest that neonates delivered via CS are more likely to experience severe respiratory distress, possibly due to the lack of labour-associated hormonal and physiological changes that prepare the neonate's lungs for breathing [13]. This finding emphasizes the need for careful monitoring and proactive respiratory support in neonates delivered by CS.

A central focus of this study was the prevalence of mechanical ventilation among neonates with MAS. Mechanical ventilation was required in 9.1% of the neonates, with a smaller subset (2.1%) requiring high-frequency oscillatory ventilation (HFOV). The need for mechanical ventilation reflects the severity of respiratory distress in MAS and underscores the importance of preparedness for advanced respiratory support in managing this condition [5]. The use of mechanical ventilation in neonates with MAS in our study aligns with findings from previous studies, where similar rates of mechanical ventilation were reported in neonates with severe MAS [14].

The study did not find significant associations between demographic factors such as gender or birth weight and the need for mechanical ventilation, suggesting that the decision to initiate mechanical ventilation is likely driven by the severity of respiratory distress rather than by demographic characteristics alone. This finding highlights the importance of clinical assessment in determining the need for mechanical ventilation in neonates with MAS.

The role of maternal and perinatal factors in MAS outcomes has been a subject of debate. Among the 329 neonates diagnosed with MAS, 10.3% were born to mothers with a history of diabetes DM, representing the most common maternal condition associated with MAS. Hypothyroidism was present in 3.6% of the cases, while both asthma and protein S deficiency were each observed in 1.8% of the mothers. Maternal hypertension was linked to 1.2% of the neonates, and maternal infection, specifically chorioamnionitis, was associated with 0.9% of the cases. These findings underscore the varied but relatively low incidence of specific maternal medical histories in neonates diagnosed with MAS, suggesting a potential, though not predominant, influence of these factors on the development of the syndrome. The low-incidence associations between these maternal conditions and MAS development in our study are intriguing, as these factors have been highlighted in previous research as potential contributors to MAS severity [11]. This discrepancy may be due to differences in population characteristics, healthcare practices, or sample size. Moreover, no significant associations were found between maternal medical history and MAS outcomes in terms of complications and mode of treatment, especially the need for mechanical ventilation, thus challenging some traditional views on the influence of maternal health on the severity of MAS [15].

Various pregnancy complications were identified among our cohort. A significant 49.2% of the cases were associated with a non-reassuring fetal heart rate, affecting 162 out of the 329 neonates. Perinatal asphyxia was present in 3.3% of the cases, involving 11 neonates. Oligohydramnios was the least common complication, associated with only 0.6% of the cases, representing two neonates. These findings highlight the importance of fetal monitoring during pregnancy, particularly the detection of a non-reassuring heart rate, as a significant proportion of MAS cases were linked to this complication.

However, the study found no significant associations between these pregnancy complications and MAS outcomes, complicating the understanding of risk factors for MAS [14]. The lack of significant associations between these factors and MAS outcomes in this study indicates a need for further research to explore the complex interplay of maternal, perinatal, and neonatal factors in MAS.

The management of MAS typically involves respiratory support tailored to the severity of the condition. In this study, 52% of the neonates required continuous positive airway pressure (CPAP) on admission, while 31.3% required non-invasive ventilation. Mechanical ventilation was necessary in 9.1% of the cases, with a subset requiring HFOV, and 3% of the neonates required inhaled nitric oxide (iNO), reflecting the need for tailored respiratory management strategies in severe cases of MAS [16,17].

Administration of bolus surfactant therapy has been shown to enhance respiratory function and improve the oxygenation index within the first six hours post-treatment in infants suffering from MAS. While this therapy decreases the severity of MAS and the need for ECMO, there is no conclusive evidence supporting its benefits in reducing mortality, air leaks, the duration of mechanical ventilation, the incidence of chronic lung disease, or intraventricular haemorrhage [18]. In our study, the administration of surfactant therapy was significantly associated with the mode of delivery, with neonates delivered via CS being more likely to receive this treatment. This finding suggests that the mode of delivery may influence the clinical course of MAS and the need for specific interventions.

Complications associated with MAS, such as PPHN and infections, were also evaluated in this study. MAS can lead to several severe complications, including chemical pneumonitis and pulmonary hypertension (PHN). Meconium functions as a chemical irritant, leading to lung injury through cytokine-mediated pathways. According to Lee et al., 23.6% of cases exhibited signs indicative of chemical pneumonitis [19]. MAS is a common cause of PHN, with an incidence ranging from 17% up to 50% in some studies [10,20]. The variation in the incidence of PHN may be influenced by the use of surfactant therapy, which is known to reduce the risk. Although air leaks are another documented complication of MAS, their occurrence varies depending on the extent of mechanical ventilation used [21].

Overall, approximately three-quarters of babies with MAS develop one or more complications, with those affected by both PHN and chemical pneumonitis experiencing the worst outcomes. However, in our study, PPHN was observed in 4.3% of the neonates, and 3.3% developed infections. These lower rates suggest a potentially milder presentation of MAS in our cohort but still underscore the importance of close monitoring and management. The length of stay in the NICU varied widely, with an average stay of 5.33 days, ranging from 2 to 44 days. This variability reflects the diverse clinical courses of MAS and the need for individualized treatment plans to optimize outcomes.

Limitations of the study

This study, while comprehensive, has several limitations that should be acknowledged. Firstly, it is a single-center study conducted at KSAFH, which may limit the generalizability of the findings to other populations and healthcare settings. Secondly, the retrospective nature of the study relies on the accuracy and completeness of medical records, which may introduce information bias. Additionally, the study did not account for potential confounding factors such as the timing of intervention and the variability in clinical management practices among healthcare providers, which could have influenced the outcomes. Finally, the study did not include long-term follow-up data, limiting the ability to assess the enduring effects of MAS and its management on neonatal health outcomes. Further multi-center, prospective studies with long-term follow-up are needed to address these limitations and provide more robust insights into the management of MAS.

## Conclusions

MAS continues to be a significant contributor to neonatal morbidity, particularly in term and post-term infants. Despite advancements in obstetric and neonatal care, MAS remains a complex condition influenced by multiple factors, including the presence of MSAF, mode of delivery, and underlying maternal and perinatal complications. The findings from this study underscore the critical need for tailored respiratory support and vigilant monitoring in neonates with MAS, particularly those born via CS. The association of MAS with severe outcomes like PPHN highlights the importance of optimizing management strategies to improve neonatal outcomes. Continued research is essential to further understand the nuanced interplay of factors contributing to MAS and to refine treatment protocols for better long-term health of affected infants.
